# The Significance of *Bacillus* spp. in Disease Suppression and Growth Promotion of Field and Vegetable Crops

**DOI:** 10.3390/microorganisms8071037

**Published:** 2020-07-13

**Authors:** Dragana Miljaković, Jelena Marinković, Svetlana Balešević-Tubić

**Affiliations:** 1 Department of Microbiological Preparations, Institute of Field and Vegetable Crops, Maksima Gorkog 30, 21000 Novi Sad, Serbia; jelena.marinkovic@ifvcns.ns.ac.rs; 2 Soybean Department, Institute of Field and Vegetable Crops, Maksima Gorkog 30, 21000 Novi Sad, Serbia; svetlana.tubic@ifvcns.ns.ac.rs

**Keywords:** *Bacillus*, biocontrol agents, antibiotics, lytic enzymes, siderophores, induced systemic resistance, plant growth-promoting rhizobacteria

## Abstract

*Bacillus* spp. produce a variety of compounds involved in the biocontrol of plant pathogens and promotion of plant growth, which makes them potential candidates for most agricultural and biotechnological applications. Bacilli exhibit antagonistic activity by excreting extracellular metabolites such as antibiotics, cell wall hydrolases, and siderophores. Additionally, *Bacillus* spp. improve plant response to pathogen attack by triggering induced systemic resistance (ISR). Besides being the most promising biocontrol agents, *Bacillus* spp. promote plant growth via nitrogen fixation, phosphate solubilization, and phytohormone production. Antagonistic and plant growth-promoting strains of *Bacillus* spp. might be useful in formulating new preparations. Numerous studies of a wide range of plant species revealed a steady increase in the number of *Bacillus* spp. identified as potential biocontrol agents and plant growth promoters. Among different mechanisms of action, it remains unclear which individual or combined traits could be used as predictors in the selection of the best strains for crop productivity improvement. Due to numerous factors that influence the successful application of *Bacillus* spp., it is necessary to understand how different strains function in biological control and plant growth promotion, and distinctly define the factors that contribute to their more efficient use in the field.

## 1. Introduction

Plant diseases, caused by various microorganisms, including fungi, bacteria, viruses, nematodes and protozoa, affect agricultural production and result in major yield losses [1]. Approximately 20–40% of losses in crop yield are caused by pathogenic infections [2]. Different strategies have been used to reduce the occurrence of plant diseases including pesticides, less susceptible cultivars, crop rotation, and other control measures, but their efficacy is usually insufficient due to the survival and resistance of soil-borne pathogens [3]. Moreover, the excessive use of synthetic pesticides has adverse effects on the environment and living organisms, and also disturbs ecosystem functioning and decreases agricultural sustainability [4].

Nowadays, research is directed to environmentally friendly alternatives for controlling plant pathogens and improving crop production, which are recommended within an integrated crop management system (ICMS) [5]. As an important component of an ICMS, biological control is defined as the use of beneficial organisms to reduce the negative effects of plant pathogens and promote positive responses by the plant [6]. The most common approach to biological control is the selection of antagonistic microorganisms, studies on their mechanisms of action and development of a biocontrol preparation [7].

*Bacillus* species are among the most investigated biocontrol agents i.e., biopesticides which contribute to suppression of plant pathogens by antagonism and/or competition [8]. Inhibition of pathogen growth by *Bacillus* spp. entails the involvement of mechanisms such as competition for nutrients and space, production of antibiotics, hydrolytic enzymes, siderophores, and/or inducing systemic resistance [9]. *Bacillus* spp. also can act as biofertilizers or biostimulators, either by facilitating the uptake of certain nutrients from the environment (nitrogen fixation, phosphate solubilization), or by providing the plant with a compound (biosynthesis of plant hormones) [10].

Hence, *Bacillus* spp. represent an alternative to plant growth enhancement agrochemicals, i.e., synthetic pesticides and fertilizers. The beneficial effects of *Bacillus* spp. on plant growth and yield have been demonstrated in several agricultural crops including wheat, maize, soybean, sunflower, common bean, tomato, pepper, potato, cucumber, and many others [11]. Application of *Bacillus* spp. to increase the productivity of field and vegetable crops is limited by variability among the results obtained in the laboratory, greenhouse and field [12]. In fact, when reintroduced by plant/soil inoculation, only 1 to 2% of rhizobacteria exert a beneficial effect on plant growth [13]. Due to numerous factors that affect the effectiveness of *Bacillus* spp., it is necessary to understand how different strains deploy biocontrol and growth-promoting actions in plants, and clearly determine the traits and manner for selecting the best acting strains [12].

This review summarizes the different mechanisms utilized by *Bacillus* spp. in plant disease control and plant growth promotion, and focuses on the new approaches to the improvement of beneficial plant–*Bacillus* interactions and *Bacillus* spp. efficiency in the field.

## 2. Genus *Bacillus*

The genus *Bacillus* was established in 1872 by Cohn and encompasses over 200 described species and subspecies belonging to the *Firmicutes* phylum. Based on the morphological characteristics, bacteria of this genus are described as rod-shaped, Gram-positive, aerobic or facultatively anaerobic, and catalase-positive [14]. Due to their broad physiological ability and capability to form endospores, *Bacillus* spp. are resistant to adverse environmental conditions and omnipresent in a wide range of habitats, including soil. *Bacillus* spp. represent the predominant soil and rhizosphere bacteria, where they comprise up to 95% of the Gram-positive bacterial populations [15]. Furthermore, they are among the most widespread endophytic bacteria [16].

*Bacillus* is a large and diverse group of non-pathogenic and pathogenic bacteria. Most species of *Bacillus*, as well as their products, are considered safe for intended use in the environment [17]. These bacteria are preferred for commercialization for their ability to secrete several bioactive metabolites, produce extremely tolerant endospores, and grow rapidly in different media [18]. Consequently, they maintain viability and can be easily formulated and stored [19]. Populations of *Bacillus* spp. can successfully persist in the soil and plant rhizosphere without any perdurable effects on other bacterial populations [20]. Commercial *Bacillus*-based preparations are developed and distributed worldwide and contain beneficial strains of *Bacillus subtilis*, *Bacillus amyloliquefaciens*, *Bacillus pumilus*, *Bacillus licheniformis*, *Bacillus megaterium*, *Bacillus velezensis*, *Bacillus cereus*, *Bacillus thuringiensis*, etc., [21].

## 3. Mechanisms of Biological Control

### 3.1. Antibiotics

The antagonistic activities of *Bacillus* spp. are frequently related to the production of secondary metabolites with antibiotic properties. These compounds mainly involve peptides with low molecular weight that are generated ribosomally (bacteriocins) or non-ribosomally (lipopeptides, peptides, polyketides).

Bacteriocins are ribosomally synthesized peptides produced by numerous bacteria which might be useful against pathogenic and antibiotic-resistant bacteria [22]. Bacteriocins act against target cells by interfering with the synthesis of the cell wall or by forming pores in the cell membrane [23]. The antimicrobial mechanisms of bacteriocins are usually directed against the species which are the same or closely related to the producers, with a narrow spectrum of action. Nevertheless, due to the production of bacteriocins, *Bacillus* spp. exhibit a broad-spectrum of antibacterial activity [24]. Some reports identify bacteriocins and bacteriocin-like substances (BLSs) (amylolysin, amysin, subtilin, subtilosin A, subtilosin B, thuricin) isolated from various *Bacillus* spp., including *B. amyloliquefaciens, B. subtilis, B. thuringiensis, B. cereus,* and *B. coagulans* [25]. Isolation and characterization of bacteriocins and BLSs may have an important use in the biological control of the pathogenic bacteria. However, *Bacillus* spp., which produce non-ribosomally synthesized lipopeptides and peptides, exhibit much stronger antimicrobial activity [26].

Cyclic lipopeptides (LPs), well known for their antagonistic action against a wide range of plant pathogens, are the most thoroughly studied antibiotic compounds in *Bacillus* spp. [27]. These peptides are synthesized by large non-ribosomal peptide synthetases (NRPSs) [28]. The primary mechanisms of LPs’ actions usually involve an interaction with the cell membrane of the target pathogens, causing changes in its structure and permeability through disruption, solubilization or formation of ion-conducting pores [26]. It has also been demonstrated that LPs interact with intracellular structures, such as DNA [29]. Recent investigations have shown that LPs influence colonization and persistence of *Bacillus* in the rhizosphere, and stimulate plant defense mechanisms [30].

The most important cyclic LPs from *Bacillus* spp. are represented by surfactin, iturin and fengycin families [27]. These antibiotics consist of amino acids, amino- or hydroxyl-fatty acids with different lengths of hydrocarbon chains. The surfactin family (surfactin, lichenysin, pumilacidin, halobacilin, bamilocyn) are heptapeptides, identified in *B. subtilis*, *B. amyloliquefaciens*, *B. licheniformis*, *B. pumilus* and *Bacillus coagulans*. Surfactins act as both antifungal and antibacterial agents. The iturin family (iturin, mycosubtilin, bacillomycin, bacillopeptins, mixirins, mojavensin, subtulene) consists of heptapeptides produced by *B. subtilis*, *B. amyloliquefaciens, Bacillus circulans, B. pumilus,* and *Bacillus vallismortis*. Iturins display inhibitory effects on a wide range of fungi, but are less active against bacteria. The fengycin family (fengycin, plipastatin, maltacin) are decapeptides of which production was detected in *B. subtilis* and *B. amyloliquefaciens*. Fengycins are useful in protecting plants against fungal pathogens [26,27,28].

Other non-ribosomally synthesized LPs include kurstakins, bacitracins, polymyxins, gramicidins, and tyrocidines. The kurstakins are cyclic or linear heptapeptides that are specific to the *B. cereus* and *B. thuringiensis*, with the ability to destabilize biological membranes of both bacteria and fungi [31]. Bacitracins are cyclic decapeptides produced by *B. licheniformis, B. subtilis*, and *B. sonorensis*, of which activity is primarily directed against Gram-positive bacteria [32]. Polymyxins are cyclic decapeptides produced by *Paenibacillus polymyxa* (*Bacillus polymyxa*), which inhibit Gram-negative bacterial cells. Gramicidins and tyrocidines are cyclic decapeptides produced by *Bacillus brevis*, active against a broad range of Gram-negative and Gram-positive bacteria [33].

Several *Bacillus* spp. are known to produce other non-ribosomally synthesized antibiotics, such as peptides (bacilysin, rhizocticin, amicoumacin, mycobacillin, diketopiperazines) and polyketides (bacillaene, dihydrobacillaene, difficidin, macrolactin), with diverse antifungal and antibacterial activities [34].

The most commonly used biocontrol agents, *B. subtilis* and *B. amyloliquefaciens*, dedicate 4–5% and 8.5% of total genetic capacity to synthesis of secondary metabolites, with the potential to produce more than two dozen structurally diverse antimicrobial compounds [27,35]. Nowadays, gene clusters encoding for bacteriocins, as well as peptides and polyketides, can be readily identified in genomic sequences by genome mining. In total, 583 putative bacteriocin gene clusters were identified from 328 strains of 57 Bacillales species, while 1231 putative non-ribosomal antimicrobial gene clusters were detected and sub-grouped into 23 types of peptide and five types of polyketide compounds distributed over 49 species of Bacillales [36].

Numerous studies revealed a broad antimicrobial effect by *Bacillus* spp. due to production of antibiotics (Table 1). *Bacillus* spp. mostly produce LPs from one family, while a few strains were identified as co-producers of different LPs [37]. Furthermore, antimicrobial activity of *Bacillus* spp. relies on the proportion and diversity in the production of antibiotics [38]. Fusarium clove rot of garlic, as well as head blight of wheat, were successfully suppressed by *B. subtilis* and/or *B. amyloliquefaciens*, due to LPs production [39,40]. In another study, *B. amyloliquefaciens* was defined as a producer of bacteriocins, surfactin, and fengycin, and was proven as a very potent biocontrol agent against numerous Gram-positive and Gram-negative bacteria, as well as *Fusarium oxysporum*, *Fusarium avenaceum*, and *Mucor* sp. [24]. Ongena et al. [41] found that iturin and fengycin produced by *B. subtilis*, which contributed to antifungal activity against *Pythium ultimum*. Han et al. [30] showed that iturin-producing *B. amyloliquefaciens* was effective in the biocontrol of *Verticillium dahliae*. Similarly, lipopeptides from *Bacillus* sp. and *B. amyloliquefaciens* such as surfactin, iturin, and fengycin, were responsible for antifungal activity against *Sclerotinia sclerotiorum* [42]. When tested for its biocontrol potential, *B. amyloliquefaciens* and *B. pumilus* LPs producing strains were very effective in the reduction in *Pseudomonas syringae* pv. *aptata* infection of sugar beet [43]. Additionally, Yang et al. [44] established that *B. subtilis* was able to suppress *Gaeumannomyces graminis* var. *tritici* infection of wheat through the production of LPs, namely iturin, surfactin, plipastatin, bacillomycin, and difficidin. Antifungal lipopeptide produced by *B. licheniformis*, determined as surfactin, was very successful against *Magnaporthe grisea*, a causative agent of rice blast [45]. Antifungal activity against *Rhizoctonia solani*, *Pythium aphanidermatum*, and *Sclerotium rolfsii* was attributed to *B. pumilus* because of the production of lipopeptide pumilacidin from the surfactin family [46].

### 3.2. Lytic Enzymes

Antimicrobial activity of *Bacillus* spp. could also be due to the production of hydrolytic enzymes such as chitinases, chitosanases, glucanases, cellulases, lipases, and proteases, which efficiently hydrolyze the major components of the fungal and bacterial cell walls. 

Chitinases (EC 3.2.1.14) are glycoside hydrolases (GHs) which degrade the β-1,4-glycosidic bonds in chitin, the second most abundant naturally available polysaccharide after cellulose, and the main component of the fungal cell wall [54]. Bacteria primarily produce chitinases in order to degrade chitin for its utilization as an energy source, whereas some bacterial chitinases are prospective biological control agents against a variety of plant diseases caused by phytopathogenic fungi [47,55]. Chitosanases (E.C. 3.2.1.132) are GHs which catalyze the hydrolytic degradation of the β-1,4-glycosidic bonds in the chitin derivative-chitosan [56]. Chitosanases are important for the extensive carbon and nitrogen recycle [57]. Since chitosan is also found in fungal cell walls, chitosanase-producing *Bacillus* spp. may be used as biocontrol agents to prevent plant infection caused by pathogens [58]. Glucanases are GHs which hydrolyze glycosidic bonds present in α-glucans and β-glucans. α-1,3-glucan is not indispensable cell wall component, but plays an important role in some fungi during cell separation and vegetative growth [59], while β-1,3-glucan is the second major component of the fungal cell wall after chitin [60]. The primary role of cell wall glucans in fungi is structural, but they may also be degraded and used as nutritional sources. *Bacillus* spp. are a rich source of α-1,3-glucanase (EC 3.2.1.84) and β-1,3-glucanase (EC 3.2.1.39). The enzymes have previously been isolated from *Bacillus brevis*, *B. licheniformis*, *B. subtilis*, *B. circulans*, and *Bacillus halodurans* [61]. Besides chitin and glucan, the skeleton of fungal cell walls contains cellulose, lipids and proteins. Bacterial cellulases, lipases and proteases may, therefore, play a significant role in the cell wall lysis that occurs during pathogen–*Bacillus* interactions [48]. 

Successful cell wall degradation depends on the activity of more than one enzyme. Chitinase activity is preceded by, or coincides with, the hydrolytic activity of other enzymes, especially glucanases. Mixtures of hydrolytic enzymes with complementary modes of action may be required for maximum efficacy, while correct combinations of enzymes may increase antifungal activity [62]. 

Recently, several reports have documented the production of lytic enzymes from *Bacillus* spp. biocontrol agents (Table 1). Chitinase-producing *B. subtilis* was effective against *Rhizoctonia solani* [47]. Crude and purified protease of *B. amyloliquefaciens* showed efficacy in biocontrol of *Fusarium oxysporum* [48]. The potential of *B. amyloliquefaciens* for biocontrol of *Clavibacter michiganensis* ssp. *michiganensis* was attributed to the production of lytic enzymes (cellulase, lipase, protease, chitinase) [49]. Hydrolytic enzymes (protease, glucanase, chitinase) produced by *Bacillus* sp. were responsible for a strong inhibitory activity against *Fusarium verticillioides* causing stalk and ear rot of maize [50]. The strength of hydrolase activity (protease, chitinase, cellulase, glucanase) was the key factor of *B. velezensis* in control of pepper gray mold caused by *Botrytis cinerea* [51]. Generally, it has been found that strains of *Bacillus* spp. which have the ability to produce cell wall hydrolases are more effective in the suppression of plant pathogens [63]. In search of efficient biocontrol agents, isolation and characterization of enzyme-producing *Bacillus* spp. should be done in order to achieve maximum survival of bacteria under detrimental environmental conditions and intrusion of pathogens [40,64].

### 3.3. Siderophores

Siderophores are metal-chelating, non-ribosomal peptides with low molecular weight produced by some microorganisms and plants, especially under iron starvation conditions [65]. Iron (Fe) is an essential element for different biological processes such as oxygen metabolism, DNA and RNA syntheses, electron transfer, and enzymatic processes. The primary role of siderophores is to chelate Fe, allowing its solubilization and extraction from minerals and organic compounds. The significance of siderophores in biological control is based on competition for Fe in order to reduce its availability for pathogens [9]. Furthermore, microbial siderophores can be reduced to donate Fe to the transport system of a plant or chelate Fe from soils, and then, do a ligand exchange with phytosiderophores, thus, providing plants with this essential element so as to enhance their growth [66]. In addition to Fe, siderophores also have the ability to bind a variety of metals in the environment, thereby acting as bioremediation agents [67].

Siderophores are grouped into three main families, depending on the functional group, including hydroxamates, catecholates, and carboxylates [9]. Most of the bacterial siderophores are catecholates, such as bacillibactin produced by several *Bacillus* spp. including *B. subtilis*, *B. amyloliquefaciens, B. cereus*, *B. thuringiensis*, etc., [68]. Besides bacillibactin, *Bacillus* spp. produce a wide variety of siderophores such as pyoverdine, pyochelin, schizokinen, petrobactin, etc., [69]. *Bacillus* spp. were better producers of siderophores than other bacterial isolates from the maize rhizosphere [70]. Siderophores produced by *Bacillus* spp. have been involved in suppression of several plant diseases (Table 1). For instance, siderophore-producing *B. subtilis* reduced the incidence of Fusarium wilt, and enhanced the growth and yield of pepper [52]. Several studies indicated synergistic antimicrobial effects of siderophores along with lipopeptides and/or lytic enzymes [42,49,50]. Similarly, *B. subtilis* is a promising biological control agent against *Bipolaris sorokiniana* due to production of siderophores, chitinase, and cellulase [53].

## 4. Systemically Induced Disease Resistance

Plants adapt to constant pathogen exposure through defense mechanisms. Resistance to pathogens, developed after proper stimulation, represents an improvement in the defense capacity of the plant. Infected plants increased their levels of signaling molecules which coordinate the activation genes for appropriate syntheses, followed by preventive structural and histological changes, preventative chemical substances (phenols and other products of secondary metabolism), and in other ways [71,72].

Plant defense mechanisms, such as induced systemic resistance (ISR), can be initiated by external agents before infection or triggered by a localized infection, resulting in systemic acquired resistance (SAR) [73]. Both biotic and abiotic factors have been used for inducing ISR in plants against different plant pathogens. ISR is promoted by non-pathogenic rhizobacteria, and is mostly dependent on the jasmonate (JA) and/or ethylene (ET) signaling pathways [74], while SAR is mediated via a salicylic acid (SA)-dependent process. SAR also activates specific sets of defense-related genes associated with the production of pathogenesis-related proteins (PR), while ISR is not accompanied by the activation of these genes [75]. The defense mediated by ISR is significantly weaker than that obtained by SAR. However, ISR and SAR together provide better protection, indicating that they can act additively in inducing resistance to pathogens [72].

Rhizobacteria promote ISR in plants through the production of various metabolites such as antibiotics, siderophores, volatile organic compounds (VOCs), and others [76]. *Bacillus* spp. are among the most studied rhizobacteria that trigger ISR in plants (Table 2), while being capable of inducing resistance against several pathogens in the same plant [77]. *B. amyloliquefaciens* induced salicylic acid-dependent resistance in tomato plants, reduced the incidence of *Tomato spotted wilt virus*, and delayed systemic accumulation of *Potato virus Y* [78]. Application of *B. cereus* significantly reduced disease incidence caused by *Botrytis cinerea* through activation of ISR [79]. Chandler et al. [80] showed that *B. subtilis* triggered ISR in rice against *Rhizoctonia solani* via jasmonic acid (JA) and ethylene (ET), as well as abscisic acid (ABA) and auxin signaling. The same authors reported an indispensable role of *B. subtilis* LPs, namely fengycin and surfactin, in the induced defense state. ISR promoting *B. amyloliquefaciens* produced VOCs and significantly reduced spot disease caused by *Xanthomonas axonopodis* pv. *vesicatoria* in pepper [81]. The ability of *B. megaterium* to reduce Septoria tritici blotch severity, caused by *Mycosphaerella graminicola*, was the result of a combination of different mechanisms, including ISR [82]. *Bacillus* endophytes of maize may protect host plants by producing antifungal lipopeptides that inhibit *Fusarium moniliforme* as well as by inducing the systemic acquired resistance [83]. 

*Bacillus* spp. can elicit ISR by inducing the synthesis of antioxidant defense enzymes. Host enzymes induced by *B. subtilis* include peroxidase (POX), polyphenol oxidase (PPO), phenylalanine ammonia-lyase (PAL), and superoxide dismutase (SOD). Increased synthesis of antioxidant defense enzymes results in ISR against early and late blight in tomato seedlings [84]. Similarly, Rais et al. [85] showed that *Bacillus* spp. enhanced the SOD, POX, PPO, and PAL activities in infected rice, thus, alleviating *Pyricularia* *oryzae*-induced oxidative damage and suppressing blast disease incidence. The antagonistic *Bacillus* sp. suppressed anthracnose disease of chili by the activation of defense-related enzymes and the accumulation of phenolic compounds [86]. Similarly, *Bacillus* sp. enhanced growth promotion and protection against *Rhizoctonia solani* and *Fusarium oxysporum* by the eliciting of defense-related enzymes (PAL, POX, PPO) in soybean [87], while *B. subtilis* was capable of impairing disease incidence, promoting seedling growth and increasing activities of antioxidant enzymes (POD, PPO, PAL) in cucumber plants [88]. The induction of resistance to *Plasmopara halstedii* by *Bacillus* sp. strain was accompanied by the accumulation of defense-related enzymes (PAL, POX, PPO) in sunflower [89].

## 5. Mechanisms of Plant Growth Promotion

### 5.1. Nutrient Availability

*Bacillus* spp. produce numerous metabolites which can increase nutrient availability to plants, and thus, directly promote plant growth and yield. Most of the plant essential nutrients are supplied through mineral fertilization, a practice which causes major economic losses, as well as posing significant problems to the environment. The use of biofertilizers which contain N_2_-fixing and/or P-solubilizing *Bacillus* spp. is a reasonable approach to reducing the negative impacts of synthetic fertilizers without compromising food safety [5,17]. N_2_-fixing and P-solubilizing *Bacillus* spp. are directly related to nutrient uptake and the subsequent growth promotion in different plants (Table 3).

Nitrogen (N) is essential for plant growth, albeit largely unavailable in its atmospheric form (more than 80%) [90]. Biological nitrogen fixation (BNF) is carried out by several groups of microorganisms that are able to absorb elemental nitrogen from the atmosphere and form compounds, which serve as plant nutrients [91]. The microorganisms produce the enzyme nitrogenase in order to catalyze the conversion of molecular dinitrogen (N_2_) to ammonia (NH_3_), which is subsequently taken by plant roots and assimilated in amino acids. BNF provides Earth’s ecosystems with about 200 million tons N per year [92]. The nitrogen-fixing microorganisms are either free-living or symbiotic. Several PGPR, including *Bacillus* spp. can decrease chemical fertilizer-N use and increase plant growth and yield through asymbiotic nitrogen fixation. BNF by rhizobacteria has been reported to contribute up to 12–70% of total N uptake in agricultural crops. The study of Kuan et al. [93] provided evidence that *B*. *pumilus* can fix atmospheric N_2_ and significantly increase the total N content and dry biomass of maize. Ding et al. [94] suggested that the *nifH* gene could be detected in both the *Bacillus* and *Paenibacillus* genera. Similarly, the study of Xie [95] reported nitrogenase activities of several *Bacillus* spp. including *B. megaterium*, *B. cereus*, *B. pumilus*, *B. circulans*, *B. licheniformis*, *B. subtilis*, *B. brevis*, and *B. firmus*. Szilagyi-Zecchin et al. [96] reported that endophytic *Bacillus* spp. were positive for the nitrogen fixation ability evaluated through the acetylene reduction assay and amplification of *nifH* gene. Increased relative abundance of *Bacillus* spp. in rice plants under the conditions of low nitrogen suggest the potential contribution of their BNF [97].

In addition to nitrogen, the plant growth directly depends on phosphorus (P). However, a high amount of P (more than 80%) is fixed in soil and is unavailable for plant uptake due to adsorption, precipitation or conversion [107]. Microorganisms that dissolve organic and inorganic phosphates belong to the group designated as Phosphate Solubilization Microorganisms (PSM) [108]. These microorganisms solubilize insoluble inorganic P and mineralize insoluble organic P [109]. Mechanisms of inorganic phosphate solubilization by microorganisms involve the production of organic and inorganic acids, siderophores, protons, hydroxyl ions, and CO_2_, which chelate cations or reduce pH in order to release P [110]. Mineralization of organic phosphate occurs due to the synthesis of extracellular enzymes such as phosphatases, phytases, and phospholipases [111]. 

Plant/soil inoculation with PSM is a promising strategy for the enhancement of plant absorption of P, while *Bacillus* spp. are among the most powerful PSM. Saeid et al. [112] showed that solubilizing exudates produced by *Bacillus* (*B. subtilis, B. megaterium, B. cereus*) are composed of gluconic, lactic, acetic, and succinic acids, confirming strong correlation between the total concentrations of organic acids and the amounts of released phosphorus. Isolates of *B. megaterium*, *B. subtilis*, and *B. simplex*, exhibited P-solubilizing ability by producing acetic, propionic, isobutyric, isocaproic, caproic, and heptanoic acids, and had positive effects on the seed germination and vegetative growth parameters of eggplant, pepper, and tomato [98]. Tao et al. [113] suggested that P-solubilization and P-mineralization could coexist in the same *Bacillus* strain. Similarly, inoculation with *B. subtilis* increased plant growth, and total accumulation of P and P uptake by cucumber plants [99].

### 5.2. Phytohormone Production

*Bacillus* spp. may directly increase plant yield through mechanisms that impart the production of phytohormones or plant growth regulators (PGRs), such as auxins, cytokinins, gibberellins, ethylene, and abscisic acid. Plant hormones are organic substances that influence the physiology and development of plants at very low concentrations. Plant hormone biosynthesis by *Bacillus* spp. has been directly related to subsequent growth promotion in different plants (Table 3).

Auxins are a group of plant hormones that stimulate plant growth, mainly through the regulation of cell division, cell elongation, and tissue differentiation. The main naturally occurring auxin is indole-3-acetic acid (IAA) [114]. Different bacteria, including *Bacillus* spp., have the ability to produce IAA and use this phytohormone to interact with plants as part of their colonization strategy, including phytostimulation and circumvention of basal plant defense mechanisms [80]. Production of IAA is widespread among soil bacteria, and approximately 80% of rhizobacteria have been estimated to produce IAA [115]. The *in vitro* application of IAA-producing *Bacillus* strains on plant roots resulted in increases in root length as well as the number of lateral roots [116]. *B. subtilis* was reported to enhance shoot and root growth, seedling vigor and leaf area of tomato, while higher levels of gibberellins and IAA were detected in treated plants [84]. Recent studies demonstrated that *Bacillus* spp. play a major role in controlling endogenous IAA levels in plant roots by regulating the auxin-responsive genes, thereby causing changes in root architecture [117].

Gibberellins (GAs) are a group of plant hormones that affect many developmental processes in higher plants, including seed germination, stem elongation, flowering, and fruiting. Gutierrez-Manero et al. [118] documented the production of gibberellins by *B. pumilus* and *B. licheniformis*. The beneficial effect of *Bacillus methylotrophicus* on plants due to the secretion of an array of gibberellins was confirmed by increasing the percentage of seed germination of lettuce, muskmelon, soybean, and vegetable mustard [100]. The same authors established that GA-producing bacterial strain increased shoot length, shoot fresh weight, leaf width, proteins, amino acids, macro and micro minerals, carotenoids and chlorophyll in lettuce.

Cytokinins (CKs) are a group of plant hormones that play a key role in promoting cell division, or cytokinesis, in plant roots and shoots. They are important regulators of other physiological and developmental plant processes such as seed germination, apical dominance, nutrient mobilization, and leaf senescence. Plants and microorganisms produce about 30 compounds from the group of CKs. It has been found that 90% of phosphate-dissolving rhizobacteria have the ability to produce CKs in vitro [119]. Arkhipova et al. [101] reported the ability of *B. subtilis* to produce CKs, while inoculation of lettuce plants increased the cytokinin content of both shoots and roots, as well as plant shoot and root weight. Ortíz-Castro et al. [102] reported that *B. megaterium* promoted the growth of *Arabidopsis thaliana* and *Phaseolus vulgaris* seedlings through CKs production. Naz et al. [103] also perceived that cytokinin-producing species, such as *Bacillus* and others, stimulated the growth of soybean plants.

Ethylene is a gaseous plant hormone that mainly regulates maturation and senescence processes, as well as response to biotic and abiotic stresses. In addition to plants, ethylene production was established in bacteria and fungi, but little has been reported on how ethylene-producing microorganisms affect plant growth. Several PGPR, including *Bacillus* spp., synthesize the enzyme 1-aminocyclopropane-1-carboxylate (ACC) deaminase that modulates ethylene levels in plants which might otherwise become growth inhibitory [120]. The enzyme ACC deaminase (3.5.99.7) cleaves ACC (direct precursor of ethylene biosynthesis in plants) into ammonia and α-ketobutyrate. Bacteria characterized by ACC deaminase activity can help maintain plant growth and development under stress conditions (drought, salt, flooding and anoxia, the presence of pathogens or contaminants) [121]. The interaction of plants with ACC deaminase-producing bacteria may be expected to promote plant growth during plant processes associated with local increase in ethylene concentration, like flower wilting or symbiosis establishment [122]. Although ACC deaminase activity has been described in many *Bacillus* strains [104,123], ACC deaminase genes (structural gene *acdS* and the regulatory gene *acdR*) could not be identified in 271 completely sequenced strains belonging to the Bacilli class, including many soil and plant-associated *Bacillus* and *Paenibacillus* species [124].

Abscisic acid (ABA) is a plant hormone with an important role in many plant physiological processes, including seed germination and stress tolerance. Park et al. [105] showed that *Bacillus aryabhattai* produced significant amounts of ABA, IAA, CKs, and GAs in culture, while inoculated soybean plants had high levels of phytohormones, longer roots and shoots, and better tolerance to heat, oxidative, and nitrosative stress. The bacterial endophyte *B. amyloliquefaciens* has been found to produce ABA and increase plant growth and resistance to salinity stress [106]. 

## 6. Efficient Use and New Approaches

### 6.1. Isolation and Identification

Prior to characterization and selection in laboratory and in greenhouse/field conditions, the search for effective strains requires isolation and identification of preferred *Bacillus* species from different sources. *Bacillus* spp. are the predominant soil, rhizosphere and endophytic bacteria [15,16]. Considering a very small proportion of beneficial microorganisms in the rhizosphere, their isolation, multiplication, and inoculation into the plant/soil trigger microbiological processes and intensify overall microbial activity [70]. Thus, only a few *Bacillus* spp. of about 200 within the genus exhibit multiple plant growth-promoting traits and might be useful in formulating inoculants [21]. Identification of isolated *Bacillus* spp. is of great importance because their beneficial traits are characteristic of certain species. Accordingly, it is necessary to use methods that can quickly and reliably test a large number of *Bacillus* spp. as the potential plant growth promoters and biological control agents.

Determination of morphological, physiological and biochemical traits is a long and often unreliable process. The most accurate method for examining the diversity of *Bacillus* spp. is their identification and characterization at the molecular level. The NCBI (National Center for Biotechnology Information) and RDP (Ribosomal Database Project) databases contain 2611 individual 16S rDNA sequences originating from 175 different species of *Bacillus*, of which only 1586 have been identified to the species level [125]. In addition to standard molecular methods such as 16S rRNA analysis, RFLP (Restriction Fragment Length Polymorphism), RAPD (Random Amplified Polymorphic DNA), rep-PCR (Repetitive element sequence-based Polymerase Chain Reaction), MLSA (Multilocus Sequence Analysis), etc., different PCR methods with species-specific primers are increasingly used for reliable differentiation of *Bacillus* spp. [126].

### 6.2. Characterization and Selection

The characterization of bacteria includes determination of numerous traits in laboratory, while selection of potential biofertilizers and biopesticides involves testing their effectiveness on the plant–soil system in greenhouse and/or field. The tests require a lot of time, which makes it impossible to examine a large number of strains. Given that no individual or combined traits can reliably predict the effectiveness in biocontrol and plant growth promotion, Akinrinlola et al. [90] suggested greenhouse pot tests as the first criterion for bacterial strain selection, instead of screening bacteria for multiple traits. According to reports, the effectiveness of *Bacillus* spp. frequently varies depending on specific plant and soil conditions, which can constrain their ability to colonize the rhizosphere and express beneficial traits [12].

The efficiency of inoculation is usually higher when bacteria are isolated from the rhizosphere of plant species, and/or soil that will be inoculated, suggesting that the growth-promoting ability of the strains is highly related to certain plant species and soil types [12]. Furthermore, the efficiency of *Bacillus* spp. from the rhizosphere is higher compared to those from the bulk soil, while both the rhizosphere and endophytic bacteria possess various beneficial traits regarding the number and the production amount of these characteristics [127]. Knowledge of biocontrol and plant growth promotion mechanisms of *Bacillus* spp. is very important for their intended use, for instance, the use of LPs and hydrolase-producing strains in the suppression of pathogen infection, or P-solubilizing and N_2_-fixing strains in P and N-deficient soils. Their efficiency as individual and combined plant/soil inoculants in different environments needs to be established through continuous selection of effective isolates in greenhouse and field trials.

### 6.3. Plant–Bacillus Interactions

Successful application of *Bacillus* spp. in the field also depends on plant–*Bacillus* interactions and it can be limited by poor colonization of the rhizosphere [128]. *Bacillus* spp. require 24 h to form a biofilm, which contributes to root colonization of *Bacillus* spp. and extends their beneficial effects in the soil [129]. Transcriptomic analysis of the *B. amyloliquefaciens* genome revealed numerous genes included in rhizosphere habituation and plant-beneficial traits, such as plant polysaccharide utilization, cell motility and chemotaxis, secondary antibiotics synthesis, and plant growth promotion-relevant clusters [130]. Gao et al. [128] demonstrated that both chemotaxis and swarming motility are important in tomato root colonization by *B. subtilis*, while the part of swarming is greater than that of chemotaxis.

However, root colonization is more effectual in indigenous strains of *Bacillus* than in laboratory or commercial strains. Emerging strategies such as microbiome engineering and breeding of microbe-optimized crops can directly or indirectly detect, modulate and enhance the traits and ways for better performance of *Bacillus* strains [3,18]. The genes involved in root colonization and plant–*Bacillus* interactions, are induced by the presence of root and seed exudates [129,130,131]. New research in plant–bacteria interactions uncovers plant capability to shape their rhizosphere and endorhiza microbiome [127]. Recent studies of the rhizobiome and the utilization of next-generation sequencing (NGS) techniques, combined with proteomics, metagenomics, metabolomics, etc., will assist to elaborate on these interactions, including how this relationship affects plant health and growth [132].

### 6.4. Bacillus-Based Preparations

In recent years, the distribution of commercial *Bacillus*-based preparations has significantly increased worldwide (Table 4). In addition to their beneficial influence on plants, effective strains of *Bacillus* spp. should be able to persist in the environment and be stable and viable for extended storage and purposeful use in the field. Resistance and stability are among the major limitations of *Bacillus*-based preparations. These bacteria are suitable for commercialization due to their ability to secrete various metabolites, produce endospores, and grow rapidly in different media [17,18,19,20]. Endospores of *Bacillus* spp. can not only endure adverse environmental conditions but survive all processing phases during production. In order to enhance sporulation and synthesis of preferable metabolites, production of *Bacillus*-based preparations should be optimized at each stage, which implies selection of appropriate strains or consortium of strains, as well as cultivation and formulation process [133].

Selection of appropriate *Bacillus* strains must be performed so as to avoid competition, especially if a preparation contains more than one species. For instance, interspecies competition between biofilms of the soil-residing bacteria *B. subtilis* and related *Bacillus* species could negatively affect their formulation and efficient use [134]. Nutrient sources such as carbon, nitrogen, inorganic salts and additional substances, as well as environmental factors such as temperature, pH value and O_2_ supply, influence growth in addition to the production of spores and metabolites in *Bacillus* species [135]. *Bacillus* spp. are suitable for preparation as either solid or liquid formulations, with the addition of different carriers, stabilizers, protectants and other supplements [133]. Further research should find the best possible production technology for each bacterial strain or bacterial combination, while taking into account the cost-effectiveness of *Bacillus*-based products. 

## 7. Conclusions

*Bacillus* spp. represent an environmentally friendly strategy for crop production improvement through different mechanisms of biological control, biofertilization and biostimulation. Although possibilities to use *Bacillus* spp. for disease incidence reduction and crop production improvement are well known, their application is not a widespread practice, mostly because of inconsistent efficiency under different conditions. The ability of *Bacillus* spp. to exhibit beneficial traits depends on the interaction of bacteria with plant and/or pathogen, and the environment. Given the great economic and ecological importance of *Bacillus* spp., it is necessary to increase the number of practically important species and find advanced methods for their rapid and comprehensive research and efficient application.

## Figures and Tables

**Table 1:** Biocontrol mechanisms exhibited by *Bacillus* species.

*Bacillus* Species	Mechanism (s)	Target Pathogen (s)	Plant Disease	Reference
*Bacillus amyloliquefaciens*	Bacteriocins, surfactin, fengycin	Various pathogenic bacteria, *Fusarium oxysporum*, *Fusarium avenaceum*, *Mucor* sp.	Several diseases of field and vegetable crops	[24]
*Bacillus amyloliquefaciens*	Iturin	*Verticillium dahliae*	Wilt of cotton	[30]
*Bacillus amyloliquefaciens*/*Bacillus subtilis*	Iturin, surfactin/surfactin, fengycin	*Fusarium graminearum*	Head blight of wheat	[39]
*Bacillus subtilis*	Surfactin, Lytic enzymes	*Fusarium* spp.	Clove rot of garlic	[40]
*Bacillus subtilis*	Iturin, fengycin	*Pythium ultimum*	Damping-off of bean	[41]
*Bacillus* sp., *Bacillus amyloliquefaciens*	Surfactin, iturin, fengycin, siderophore	*Sclerotinia sclerotiorum*	White mold of common bean	[42]
*Bacillus pumilus*, *Bacillus amyloliquefaciens*	Lipopeptides	*Pseudomonas syringae* pv. *aptata*	Leaf spot disease of sugar beet	[43]
*Bacillus subtilis*	Iturin, surfactin, plipastatin, bacillomycin, difficidin	*Gaeumannomyces graminis* var. *tritici*	Take-all of wheat	[44]
*Bacillus licheniformis*	Surfactin	*Magnaporthe grisea*	Blast disease of rice	[45]
*Bacillus subtilis*	Chitinase	*Rhizoctonia solani*	Stem canker and black scurf of potato	[47]
*Bacillus amyloliquefaciens*	Protease	*Fusarium oxysporum* f. sp. *lycopersici*	Wilt disease of tomato	[48]
*Bacillus amyloliquefaciens*	Siderophores, cellulase, lipase, protease, chitinase	*Clavibacter michiganensis*	Bacterial canker of tomato	[49]
*Bacillus* sp.	Protease, glucanase, chitinase, siderophores	*Fusarium verticillioides*	Stalk and ear rot of maize	[50]
*Bacillus velezensis*	Protease, Chitinase, Cellulase, Glucanase	*Botrytis cinerea*	Gray mold disease of pepper	[51]
*Bacillus subtilis*	Siderophores	*Fusarium oxysporum* f.sp. *capsici*	Wilt of pepper	[52]
*Bacillus subtilis*	Siderophores, lytic enzymes	*Bipolaris sorokiniana*	Spot blotch of wheat	[53]

**Table 2:** Induced systemic resistance elicited by *Bacillus* species.

*Bacillus* Species	Target Pathogen (s)	Plant Disease	Reference
*Bacillus amyloliquefaciens*	*Tomato spotted wilt virus, Potato virus Y*	Wilt disease of tomato	[78]
*Bacillus cereus*	*Botrytis cinerea*	Gray mold disease of field and vegetable crops	[79]
*Bacillus subtilis*	*Rhizoctonia solani*	Sheath blight of rice	[80]
*Bacillus amyloliquefaciens*	*Xanthomonas axonopodis* pv. *vesicatoria*	Leaf spot disease of pepper	[81]
*Bacillus megaterium*	*Mycosphaerella graminicola*	Septoria tritici blotch of wheat	[82]
*Bacillus subtilis*, *Bacillus amyloliquefaciens*	*Fusarium moniliforme*	Ear, stalk, and root rots of maize	[83]
*Bacillus subtilis*	*Alternaria solani*, *Phytophthora infestans*	Early and late blight of tomato	[84]
*Bacillus* spp.	*Pyricularia oryzae*	Blast disease of rice	[85]
*Bacillus* sp.	*Colletotrichum capsica*	Anthracnose of chili	[86]
*Bacillus* sp.	*Rhizoctonia solani*, *Fusarium oxysporum*	Root rot and wilt of soybean	[87]
*Bacillus subtilis*	*Fusarium oxysporum* f. sp. *cucumerinum*	Root rot of cucumber	[88]
*Bacillus* sp.	*Plasmopara halstedii*	Downy mildew of sunflower	[89]

**Table 3:** Plant growth-promoting mechanisms exhibited by *Bacillus* species.

*Bacillus* Species	Mechanism (s)	Treated Plant (s)	Effect	Reference
*Bacillus pumilus*	N_2_-fixation	Maize	Increase the total N content and dry biomass	[93]
*Bacillus* sp.	N_2_-fixation	Maize	Increased seed germination and root volume	[96]
*B. megaterium*, *B. subtilis*, *B. simplex*	P-solubilization	Eggplant, pepper, tomato	Promoted seed germination and vegetative growth	[98]
*Bacillus subtilis*	P-solubilization	Cucumber	Increased plant growth, total accumulation of P and P uptake	[99]
*Bacillus subtilis*	IAA, GA	Tomato	Enhanced shoot and root growth, seedling vigor and leaf area, higher levels of hormones	[84]
*Bacillus methylotrophicus*	GAs	Lettuce	Increased shoot length, shoot fresh weight, leaf width, proteins, amino acids, macro and micro minerals, carotenoids and chlorophyll a	[100]
*Bacillus subtilis*	CKs	Lettuce	Increased plant shoot and root weight, higher CKs levels	[101]
*Bacillus megaterium*	CKs	Common bean	Promoted growth of seedlings	[102]
*Bacillus* spp.	IAA, CKs, GAs, ABA	Soybean	Better growth and higher proline contents	[103]
*Bacillus subtilis*	IAA, ACC deaminase	Tomato	Increased shoot and root biomass and chlorophyll (a and b) contents	[104]
*Bacillus aryabhattai*	ABA, IAA, CKs, GAs	Soybean	Longer roots and shoots, higher hormone levels, better stress tolerance	[105]
*Bacillus amyloliquefaciens*	ABA	Rice	Increased growth and stress tolerance	[106]

**Table 4:** Examples of commercial *Bacillus*-based preparations.

*Bacillus* Species	Preparation	Plant (s)	Company
*Bacillus subtilis*	Serenade^®^	Vegetables, fruits	AgraQuest Inc., USA
*Bacillus subtilis*	Companion^®^	Legumes, vegetables, maize, and others	Growth Products Ltd., USA
*Bacillus subtilis*	Kodiak^®^	Legumes, cotton, and others	Gustafson Inc., USA
*Bacillus subtilis*	Cease^®^	Several crops	BioWorks Inc., USA
*Bacillus subtilis*	Subtilex^®^	Vegetables, legumes, cotton, and others	Becker Underwood, Inc., USA
*Bacillus subtilis*	Pro-Mix^®^	Soybean, ornamentals, and others	Premier Horticulture Inc., Canada
*Bacillus subtilis*	FZB24^®^	Several crops	ABiTEP GmbH, Germany
*Bacillus subtilis*	Bio Safe^®^	Legumes, vegetables, cotton	Lab. Biocontrole Farroupilha, Brazil
*Bacillus subtilis*	Ecoshot^®^	Vegetables, legumes, fruits, and others	Kumiai Chemical Industry, Japan
*Bacillus subtilis*	Biosubtilin^®^	Cereals, vegetables, legumes, oilseeds, cotton, and others	Biotech International Ltd., India
*Bacillus amyloliquefaciens*	BioYield^®^	Legumes, vegetables, tobacco	Gustafson Inc., USA
*Bacillus amyloliquefaciens*	Rhizocell GC^®^	Cereals, sugar beet	Lallemand Plant Care, France
*Bacillus amyloliquefaciens*	RhizoVital^®^42, RhizoVital^®^42TB	Vegetables, cereals, ornamentals	ABiTEP GmbH, Germany
*Bacillus pumilus*	Ballad^®^ Plus	Cereals, oilseeds, sugar beet, sweet corn	AgraQuest Inc., USA
*Bacillus pumilus*	Yield Shield^®^	Legumes, cereals, vegetables, sugar beet, cotton	Bayer CropScience, USA
*Bacillus pumilus*	Sonata^®^	Vegetables, fruits	AgraQuest Inc., USA
*Bacillus* *licheniformis*	EcoGuard^®^	Several crops	Novozymes A/S Denmark, Novozymes Biologicals, USA
*Bacillus velezensis*	Botrybel^®^	Vegetables, fruits	Agricaldes, Spain
*Bacillus megaterium*	Symbion-P^®^	Cereals, legumes, oilseeds, vegetables	T. Stanes & Co. Ltd., India
*Bacillus* sp.	Sublic^®^	Several crops	ELEP Biotechnologies, Italy
*Bacillus* spp.	*Bacillus* SPP^®^	Several crops	Bio Insumos Nativa, Chile
